# Severe Hyperkalemia and Bilateral Adrenal Metastasis

**DOI:** 10.1155/2009/831979

**Published:** 2010-03-02

**Authors:** Michael Nagler, Beat Müller, Verena Briner, Ralph Winterhalder

**Affiliations:** ^1^Department of Medicine, Luzerner Kantonsspital, 6000 Luzern 16, Switzerland; ^2^Division of Medical Oncology, Luzerner Kantonsspital, 6000 Luzern 16, Switzerland

## Abstract

Adrenal metastases are a common finding in metastatic lung and breast cancer. Often there are no clinical symptoms suggesting them. In this paper, we present a case of a 66-year-old man with metastatic lung cancer suffering from severe hyperkaliemia due to hypoaldosteronism as a result of bilateral adrenal metastasis.

## 1. Introduction

Hyperkalemia most often is caused by potassium retention due to renal insufficiency. A rare cause of hyperkalemia is adrenal insufficiency induced by adrenal metastasis. The latter is found in metastatic lung cancer in up to 40 to 60 percent, however in the majority of patients without clinical significance.

## 2. Case Report

A 66-year-old man was admitted to the hospital due to symptomatic bradycardia and a progressive generalized muscular weakness starting two days earlier. He had a history of metastatic nonsmall lung cancer (type adenocarcinoma), since 15 months. In addition coronary artery disease was known for two years. Initial chemotherapy with carboplatin and gemcitabine was initiated and transiently a good tumor response was achieved. The primary tumor and the mediastinal lymph nodes progressed and thus treatment was switched to a tyrosine kinase-inhibitor therapy with erlotinib. Due to side effects (muscular weakness of the limbs), the therapy was discontinued fourteen weeks later although having stabilized the disease and the symptoms resolved completely.

At the actual presentation physical examination revealed a bradycardia (40/min), normal blood pressure, and tetraparesis. The cranial nerves responded normal. The skin color was tanned even at sites not being exposed to the sunlight.

Laboratory analysis demonstrated severe hyperkalemia of 8.8 mmol/L, mild hyponatremia (132 mmol/L), and metabolic acidosis (pH 7.2) found. A rise in creatinine (127 *μ*mol/L) and serum urea (21.4 mmol/L) level was seen for the first time. The electrocardiogram confirmed sinusbradycardia with broad QRS complexes.

**Figure 1:**
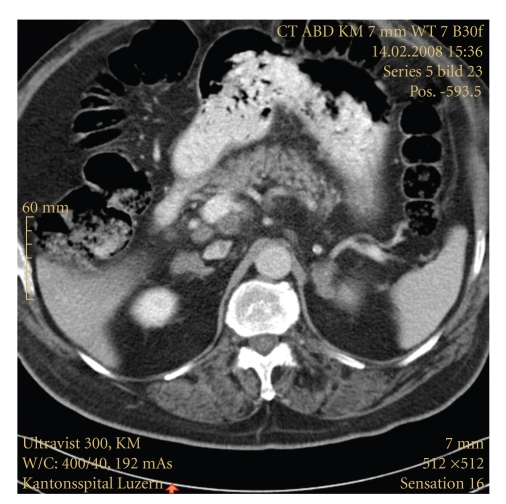


No obvious cause for hyperkalemia was present (e.g., chronic renal insufficiency, potassium release from cells due to rhabdomyolysis or tumor lysis) a low dose ACTH stimulation test [1] revealed an insufficient cortisone response (184 nmol/L). A low aldosterone level (0.10 nmol/L) and an elevated plasma renin activity (33.7 mU/L) confirmed the initial hypothesis of hypoaldosteronism. Since the patient had no obvious cause for hypoadrenalism (such as long-term corticosteroid therapy, infection, autoimmune disorder), we suggested the adrenal metastases shown by a computed tomography scanning (Figure 1) being the cause for adrenal insufficiency.

After substitution of fluids and hydrocortisone as well as administration of insulin and glucose, sodium bicarbonate, salbutamol, furosemide, and cation exchange resin, the patient improved rapidly. The tetraparesis disappeared totally, and heart rate rose to normal. Serum potassium concentration and pH level normalized and stayed stable under hydrocortisone administration.

## 3. Discussion

The prevalence of Addison's disease has been estimated at 35 to 120 per million [2–4]. At the time when Thomas Addison in 1855 reported about the “disease of the suprarenal capsules” most of the cases were caused by disseminated tuberculosis [5]. Today autoimmune adrenalitis counts for 70–90 percent of the cases, tuberculosis is responsible for 7–20 percent and the residual is caused by adrenal hemorrhage or infarction, drugs, and suppression by metastatic cancer [6, 7] or lymphoma [8–11]. Autopsy studies suggest infiltration of the adrenal glands by metastatic cancer being a common finding. It is demonstrated in up to 40–60 percent of patients with disseminated lung or breast cancer [6, 7], probably because of their rich blood supply. Apparently clinical adrenal insufficiency is rarely reported though there are few reports about Addisonian crisis due to metastatic adrenal infiltration by lung cancer or lymphoma [12–15]. Some cases might be missed because other causes are explaining reduced general conditions and hyperkalemia such as impaired renal function and potassium sparing diuretics. Unfortunately there are only unimpressive signs and symptoms suggesting adrenal insufficiency. Typically patients present with generalised weakness, fatigue, gastrointestinal complaints, dehydration, postural hypotension, and—in case of adrenal crisis—shock [16]. Characteristic is a hyperpigmentation, which is evident in nearly all patients with primary adrenal insufficiency [17]. Hyponatremia is found in 85 percent of patients, and hyperkalemia often associated with a mild hyperchloremic acidosis occurs in 60 percent of cases.

## 4. Learning Point

In patients with metastatic lung cancer adrenal metastasis is frequent however symptoms are rare. However, hyperkalemia may be the result of adrenal insufficiency due to adrenal metastasis, which can easily be revealed by low-dose ACTH testing.

## References

[1] Henzen C, Suter A, Lerch E, Urbinelli R, Schorno XH, Briner VA (2000). Suppression and recovery of adrenal response after short-term, high-dose glucocorticoid treatment. *The Lancet*.

[2] Willis AC, Vince FP (1997). The prevalence of Addison’s disease in Coventry, UK. *Postgraduate Medical Journal*.

[3] Laureti S, Vecchi L, Santeusanio F, Falorni A (1999). Is the prevalence of Addison’s disease underestimated?. *Journal of Clinical Endocrinology and Metabolism*.

[4] Kong M-F, Jeffcoate W (1994). Eighty-six cases of Addison’s disease. *Clinical Endocrinology*.

[5] Addison T (1855). *On the Constitutional and Local Effects of Disease of the Supra-Renal Capusles*.

[6] Lam K-Y, Lo C-Y (2002). Metastatic tumours of the adrenal glands: a 30-year experience in a teaching hospital. *Clinical Endocrinology*.

[7] Cedermark BJ, Blumenson LE, Pickren JW (1977). The significance of metastases to the adrenal glands in adenocarcinoma of the colon and rectum. *Surgery Gynecology and Obstetrics*.

[8] Nomura K, Demura H, Saruta T (1994). Addison’s disease in Japan: characteristics and changes revealed in a nationwide survey.. *Internal Medicine*.

[9] Irvine WJ, Barnes EW (1972). Adrenocortical insufficiency. *Clinics in Endocrinology and Metabolism*.

[10] Zelissen PMJ, Bast EJ, Croughs RJM (1995). Associated autoimmunity in Addison’s disease. *Journal of Autoimmunity*.

[11] Kasperlik-Zaluska AA, Migdalska B, Czarnocka B, Drac-Kaniewska J, Czech W, Niegowska E (1991). Association of Addison’s disease with autoimmune disorders—a long-term observation of 180 patients. *Postgraduate Medical Journal*.

[12] Serrano S, Tejedor L, Garcia B, Hallal H, Polo JA, Alguacil G (1993). Addisonian crisis as the presenting feature of bilateral primary adrenal lymphoma. *Cancer*.

[13] Huminer D, Garty M, Lapidot M, Leiba S, Borohov H, Rosenfeld JB (1988). Lymphoma presenting with adrenal insufficiency. Adrenal enlargement on computed tomographic scanning as a clue to diagnosis. *American Journal of Medicine*.

[14] Akcay MN, Tekin SB, Akcay G (2003). Addisonian crisis due to adrenal gland metastasis in Hodgkin’s disease. *International Journal of Clinical Practice*.

[15] Munoz A, Onate J, Mane JM (2001). Addisonian crisis as first manifestation of adrenal gland insufficiency in patient diagnosed with lung cancer. *Anales de Medicina Interna*.

[16] Burke CW (1985). Adrenocortical insufficiency. *Clinics in Endocrinology and Metabolism*.

[17] Barnett AH, Espiner EA, Donald RA (1982). Patients presenting with Addison’s disease need not be pigmented. *Postgraduate Medical Journal*.

